# Magnetic SERS Strip Based on 4-mercaptophenylboronic Acid-Modified Fe_3_O_4_@Au for Active Capture and Simultaneous Detection of Respiratory Bacteria

**DOI:** 10.3390/bios13020210

**Published:** 2023-01-31

**Authors:** Jingfei Li, Jin Chen, Yuwei Dai, Zhenzhen Liu, Junnan Zhao, Shuchen Liu, Rui Xiao

**Affiliations:** 1 Beijing Institute of Microbiology and Epidemiology, Beijing 100071, China; 2 Department of Clinical Laboratory, Beijing Ditan Hospital, Capital Medical University, Beijing 100015, China; 3 Beijing Institute of Radiation Medicine, Beijing 100850, China

**Keywords:** Fe_3_O_4_@Au, lateral flow assay, respiratory bacteria, SERS, 4-mercaptophenylboronic acid

## Abstract

The rapid diagnosis and detection of respiratory bacteria at the early stage can effectively control the epidemic spread and bacterial infection. Here, we designed a rapid, ultrasensitive, and quantitative lateral flow immunoassay (LFA) strip for simultaneous detection of respiratory bacteria *S. aureus* and *S. pneumoniae*. In this assay, the surface enhanced Raman scattering (SERS) tags were designed through combining magnetite Raman enhancement nanoparticle Fe_3_O_4_@Au/DTNB and recognition element 4-mercaptophenylboronic acid (4-MPBA). Further, 4-MPBA could capture multiple bacteria in a complex environmental solution. Based on the strategies, Fe_3_O_4_@Au/DTNB-mediated magnetic enrichment and 4-MPBA-mediated universal capture capabilities improved the detection sensitivity, the limits of detection for *S. aureus* and *S. pneumoniae* were as low as 12 and 11 CFU mL^−1^, respectively, which were more sensitive than those of colloidal gold method. The Fe_3_O_4_@Au/DTNB/Au/4-MPBA-LFA also exhibited good reproducibility, excellent specificity, and high recovery rates in sputum samples, indicating its potential application in the detection of respiratory bacteria samples.

## 1. Introduction

Common respiratory bacteria, mainly including *Staphylococcus aureus*, *Escherichia coli*, *Streptococcus pneumoniae*, and *Pseudomonas aeruginosa* [1], infections caused by these bacteria can induce serious respiratory diseases [2]. Accurate and sensitive respiratory bacteria detection is crucial to controlling bacterial infections and human health. Numerous analytical methods for pathogen detection have been developed, conventional detection method standard plate colony counting generally takes several days and the sensitivity is low [3]. Common molecular biological detection methods, such as polymerase chain reaction (PCR) [4], enzyme-linked immunosorbent assay (ELISA) [5], surface plasmon resonance [6], and nucleic acid hybridization [7], however, all these methods typically have the disadvantages of time-consuming, laborious sample pretreatment, and expensive equipment requirements, which limit their application of bacterial detection [8,9,10]. Therefore, a rapid and ultrasensitive detection technology for respiratory pathogens is highly required.

SERS has been used in various biomarker or pathogens detection field owing to its high sensitivity, specificity, and high speed [11]. In particularly, SERS can provide spectroscopic fingerprint and nondestructive data acquisition even in complex sample environment with fewer background interference [12]. For instance, Zhang has reported a potential biomarker exosomal Programmed cell death 1 ligand 1 (PD−L1) for immunotherapy and provided SERS detection platform for the trace detection of clinical cancers, the detection limit for PD−L1 and exosomes based on PD−L1 were 0.1 ng mL^−1^ and 4.8 × 10^6^ particles mL^−1^, respectively [13]. Cheng has fabricated SERS-based sensor based on Au-Ag nanocomplex-decorated ZnO nanopillars on paper for direct serological detection of liver diseases within 1 min [14]. Yang has reported a novel SERS strategy based on three-dimensional (3D) DNA walker for quantitative analysis of *Salmonella typhimurium* with a superior limit detection of 4 CFU mL^−1^ [15]. Lateral flow immunoassay (LFA) is a popular point-of-care testing (POCT) detection technology [16], which is simple operation, short analysis time, and low cost [17]. However, conventional LFA is mainly based on color visualization using colloidal gold as signal reporters, thus providing low sensitivity and only half-quantification detection ability [18]. SERS-based LFA strips were used for pathogen detection because of ultrasensitive and quantitative analysis capabilities, which mainly rely on the SERS tags consisting of a SERS-enhanced substrate, Raman reporters, and targeting biomolecules [19]. Recently, magnetic nanomaterials, such as Au-coated Fe_3_O_4_ magnetic nanoparticles (Fe_3_O_4_@Au MNPs), have been used in the detection of complex samples due to their excellent versatility and stability [20]. Therefore, after Fe_3_O_4_@Au MNPs are introduced into the biosensors, the magnetic SERS-based system can be endowed with magnetic enrichment capability in the complex environmental solutions, which avoid sample pretreatment process and shorten detection time.

Recognition elements are critical for the detection performance of target pathogen in typical SERS biosensor systems. The recognition elements such as antibody, aptamers, and antibiotic are used to graft on SERS tags to capture target pathogen [21]. Among them, antibodies are obtained from immunized animals, which are expensive and poor stability [22]. The screening process of antibodies is time-consuming, especially, the antibodies are lacking and difficult to prepare or screen in a short time for rare and sudden bacterial infection. The optimization process of aptamers is also difficult, and the binding between target bacteria and aptamers generally needs high-salt ions and a relatively long time [23,24]. A pair of antibody or aptamer is used to detect only one target bacterium. Antibiotics usually lack a high affinity for pathogens. Currently, 4-mercaptophenylboronic acid (4-MPBA) as a bacterial capture molecule is used in the field of biosensor detection. It can bind peptidoglycan on the cell walls of bacteria and thus capture various kinds of bacteria simultaneously, avoiding the process of screening antibodies or aptamers and possessing high affinity for the target bacteria [25,26,27], which appears to be a promising candidate and achieves broad-spectrum bacteria capture capability.

In this study, the magnetic SERS tags Fe_3_O_4_@Au/DTNB/Au/4-MPBA were fabricated using Fe_3_O_4_@Au MNPs as the substrate and 4-mercaptophenylboronic acid (4-MPBA) as Raman recognition element. The high-performance Fe_3_O_4_@Au/DTNB/Au/4-MPBA SERS tags possessed good stability, universal capture, magnetic enrichment, and excellent SERS activity. Based on these advantages, our results showed the bacterial capture efficiency of SERS tags was about 95.4% even at a low concentration and the limits of detection (LODs) were as low as 12 and 11 CFU mL^−1^ for respiratory bacteria *S. aureus* and *S. pneumoniae*, respectively.

## 2. Experimental

### 2.1. Materials and Chemicals

Phosphate buffered saline (PBS, 0.01 M, pH 7.4), polyethyleneimine (PEI), trisodium citrate dihydrate, polyvinylpyrrolidone (PVP, 40 kDa), hydroxylamine hydrochloride, 4-mercaptophenylboronic acid (4-MPBA), chloroauric acid tetrahydrate (HAuCl_4_·4H_2_O), fetal bovine serum (FBS), Tween-20, 5,5′-Dithiobis-(2-nitrobenzoic acid) (DTNB), and crystal violet (CV) were obtained from Sigma-Aldrich (St. Louis, MO, USA). Mouse monoclonal antibodies (Catalog # MA1-10708, MA1-10709) were obtained from Thermo Fisher (Shanghai, China). Rabbit monoclonal antibodies (Catalog # Strpcpnu-001, Strpcpnu-002) were purchased from Xinxin Bio, Ltd. (Changzhou, China). Nitrocellulose (NC) membranes (Prima40 with 18 μm pore size, CN140 with 8 μm pore size, and CN95 with 15 μm pore size) were purchased from Sartorius (Gottingen, Germany), plastic backing card, sample loading pad, and absorbent pad were obtained from Jieyi Biotechnology Co., Ltd. (Shanghai, China). All analytical grade reagents were purchased from Sigma-Aldrich (USA).

### 2.2. Instruments

Transmission electron microscope (TEM) images were captured by a Hitachi H-7650 TEM (Tokyo, Japan) at 50 kV. High resolution transmission electron microscope (HRTEM) images were captured using the FEI Tecnai G2 F20 electron microscopy (Hillsboro, OR, USA) at 200 kV. Scanning electron microscope (SEM) images were obtained by a JEOL JSM-7001F instrument (Tokyo, Japan) at an operating voltage of 10 kV. Zeta potential data were tested by a Malvern Nano-ZS90 ZetaSizer (Malvern, UK) instrument. Raman spectra data were collected by a portable Raman system (B&W Tek, i-Raman Plus BWS465-785H spectrometer, Plainsboro, NJ, USA).

### 2.3. Fabrication of Fe_3_O_4_@Au/DTNB/Au/4-MPBA Tags

**Scheme 1:**
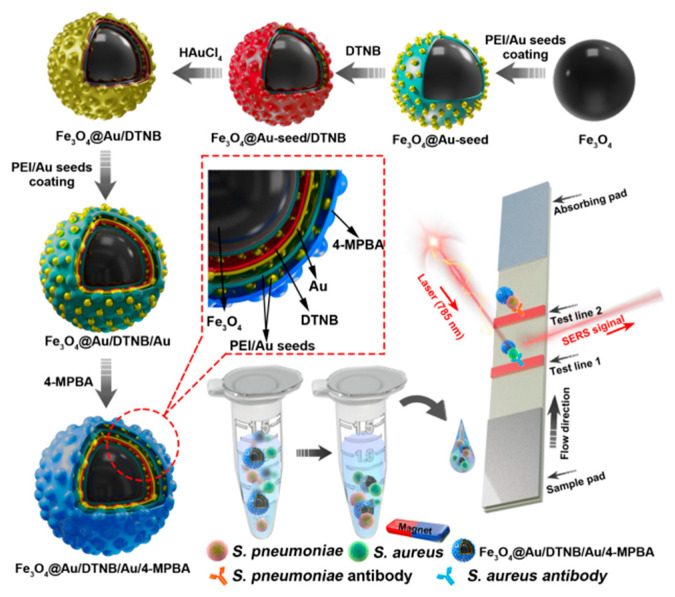
Fabrication of Fe_3_O_4_@Au/DTNB/Au/4-MPBA and schematic diagram of Fe_3_O_4_@Au/DTNB/Au/4-MPBA SERS strip for detecting two respiratory bacteria.

The design route of Fe_3_O_4_@Au/DTNB/Au/4-MPBA tags was demonstrated in Scheme 1. Fe_3_O_4_@Au/DTNB was fabricated by previously reported PEI-mediated seed growth method [28]. The specific synthesis details were provided in the Supporting Information S1. Fe_3_O_4_@Au/DTNB was dispersed in the PEI aqueous solution (2 mg mL^−1^) followed by sonication for 0.5 h and washed for three times, then mixed with colloidal 3-nm AuNPs and sonicated for another 0.5 h to obtain Fe_3_O_4_@Au/DTNB/Au. Finally, 200 μL of 4-MPBA (10 mM) was added to the prepared Fe_3_O_4_@Au/DTNB/Au and continuously sonicated for 2 h to fabricate Fe_3_O_4_@Au/DTNB/Au/4-MPBA tags. The Fe_3_O_4_@Au/DTNB/Au/4-MPBA tags were stored at room temperature for further use.

### 2.4. Fabrication of Lateral Flow Strip Bacteria Detection System

A LFA strip was prepared with absorbent pad, sample pad, and nitrocellulose membrane (NC membrane). *S. aureus* (0.6 mg mL^−1^) and *S. pneumoniae* (0.4 mg mL^−1^) antibodies were sprayed onto NC membrane as test line 1 (T1) and test line 2 (T2), respectively. NC membrane was placed at 37 °C for 2 h. Subsequently, absorption pad and sample pad were assembled onto the both ends of NC membrane to ensure liquid flow. The prepared card was then cut into the strips with a width of 3 mm and stored in a vacuum desiccator for further use.

### 2.5. Preparation of Bacterial Sample

Bacteria concentrations, including *S. aureus* and *S. pneumoniae*, were verified by plate counting method [29]. The bacteria were cultured using 5% sheep blood agar plates at 37 °C overnight. The initial bacterial solution was obtained by several bacterial colonies on the plates picked into 0.5 mL of sterile PBS solution. The initial solution was then diluted 10^4^ to 10^6^ times using PBS solution, 0.1 mL of diluted bacterial suspensions were withdrawn and dropped on blood agar plates followed by incubation at 37 °C overnight. The colony forming units (CFUs) on the plates were counted to calculate the concentration of initial bacterial solution, based on the calculated results, the testing concentration of 10^8^ cells mL^−1^ was prepared for further use.

### 2.6. Detection of Respiratory Bacteria Using Fe_3_O_4_@Au/DTNB/Au/4-MPBA-Based LFA Strip

The detection of *S. aureus* and *S. pneumoniae* was conducted in a 2 mL tube. First, 4 μL of liquid Fe_3_O_4_@Au/DTNB/Au/4-MPBA tags was added into 1 mL of each bacteria suspension and magnetically collected after incubation for 20 min. The bacteria suspension in the tube was removed and 80 μL of PBS running buffer was added followed by ultrasonic for 1 min, the prepared strip was inserted into the tube for 20 min, then the strip was dried and SERS intensities of test lines were tested with a portable Raman spectrometer under 785 nm laser excitation.

### 2.7. Detection of Respiratory Pathogens S. aureus and S. pneumoniae in Biological Samples

The recovery rates (%) of bacteria were calculated using sputum specimens. The bacterial concentrations of 10^6^ CFU mL^−1^ and 10^3^ CFU mL^−1^ were respectively spiked to the diluted sputum specimens, the specific operation was conducted according to the protocol in Section 2.6. The recovery rates were determined according to the previously established calibration curves and the tests were conducted in triplicate for each sample.

## 3. Results and Discussion

### 3.1. Principle of Fe_3_O_4_@Au/DTNB/Au/4-MPBA-Based LFA Strip for the Simultaneous Detection of S. aureus and S. pneumoniae

In this detection system, Fe_3_O_4_@Au/DTNB/Au/4-MPBA was used as SERS tags to simultaneously capture two target bacteria and improve the sensitivity of LFA. Fe_3_O_4_@Au/DTNB/Au/4-MPBA tags presented good dispersity, magnetic responsiveness, universal capture, and strong SERS activity in a complex solution. The operating procedure of the simultaneous detection of respiratory bacteria was displayed in Scheme 1. The detection principle was based on antibody-antigen interaction. First, Fe_3_O_4_@Au/DTNB/Au/4-MPBA tags were added into the solution containing *S. aureus* and *S. pneumoniae* followed by shaking for about 20 min. The target bacteria were captured by Fe_3_O_4_@Au/DTNB/Au/4-MPBA tags during incubation, the Fe_3_O_4_@Au/DTNB/Au/4-MPBA-bacteria complexes were separated from the bacteria solution using a magnet, then resuspended in the prepared running buffer solution and ultrasonic for several seconds. Finally, LFA strips were inserted into the running buffer solution, the complexes Fe_3_O_4_@Au/DTNB/Au/4-MPBA-bacteria slowly flowed along the strip from the sample loading pad to the absorbent pad under capillary forces. Due to the interaction between antibody and antigen, the complexes were captured by the *S. aureus* and *S. pneumoniae* antibodies pre-coated on the T1 and T2 lines, respectively, and then the sandwich-like immunocomplexes Fe_3_O_4_@Au/DTNB/Au/4-MPBA-bacteria-detection antibodies were formed at the test lines. There was no control line (C line) on NC membrane owing to lack 4-MPBA antibody. Although C line was used to evaluate whether the tags and NC membrane were effective, our study revealed that the target bacteria were captured by fabricated Fe_3_O_4_@Au/DTNB/Au/4-MPBA tags, then the Fe_3_O_4_@Au/DTNB/Au/4-MPBA-bacteria complexes were captured by the antibodies on NC membranes, our results demonstrated the tags possessed excellent capture performance and the immune binding with antibodies on the test lines was normal, which further indicated that the prepared NC membranes were available and our detection system was successfully fabricated, relevant studies of lack of C line on NC membrane have also been reported [30,31]. Moreover, the Raman signal intensities became higher with the increase of tags and bacteria, which provided the quantification detection method, after reaction for 20 min, two dark bands appeared on the strip in the presence of *S. aureus* and *S. pneumoniae*, only one dark band (T1 line or T2 line) was observed in the presence of *S. aureus* or *S. pneumoniae*, while no line was observed in absent of bacteria, which indicated that the detection system was successfully fabricated. Finally, Raman intensities were measured by a portable Raman spectrometer.

### 3.2. Characterization of Fe_3_O_4_@Au/DTNB/Au/4-MPBA Tags

**Figure 1:**
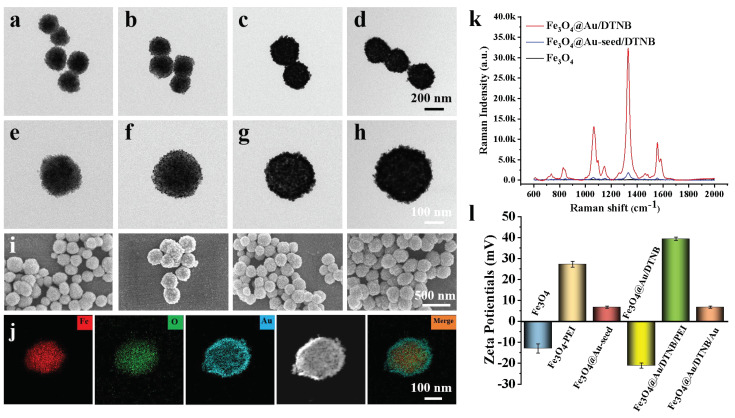
Structure characterization of Fe_3_O_4_@Au/DTNB/Au/4-MPBA. TEM images of (**a**) Fe_3_O_4_, (**b**) Fe_3_O_4_@Au-seed/DTNB, (**c**) Fe_3_O_4_@Au/DTNB, (**d**) Fe_3_O_4_@Au/DTNB/Au/4-MPBA tags, and their corresponding magnified TEM images in (**e**), (**f**), (**g**), and (**h**), respectively. (**i**) SEM images of (**a**–**d**). (**j**) EDS elemental mapping images of Fe_3_O_4_@Au/DTNB/Au/4-MPBA. (**k**) SERS signal intensities of Fe_3_O_4_, Fe_3_O_4_@Au-seed/DTNB, and Fe_3_O_4_@Au/DTNB. (**l**) Zeta potentials of Fe_3_O_4_, Fe_3_O_4_−PEI, Fe_3_O_4_@Au-seed, Fe_3_O_4_@Au/DTNB, Fe_3_O_4_@Au/DTNB/PEI, and Fe_3_O_4_@Au/DTNB/Au.

As shown in Figure 1a,e, Fe_3_O_4_ MNPs were successfully fabricated and the diameter was about 200 nm [32]. Au seeds adhered to the surface of Fe_3_O_4_ after 3-nm AuNPs were coated on synthetic Fe_3_O_4_−PEI (Figure 1b,f). A rough Au shell of Fe_3_O_4_@Au/DTNB with a size of 15 nm was decorated outside the Fe_3_O_4_ core under the reduction of HAuCl_4_ (Figure 1g) and the obtained Fe_3_O_4_@Au/DTNB was uniform with a diameter of approximately 280 nm (Figure 1c). Au seeds in Fe_3_O_4_@Au/DTNB/Au were observed again after the second layer of 3-nm AuNPs were modified with Fe_3_O_4_@Au/DTNB (Figure 1d,h). Moreover, SEM images further demonstrated that Fe_3_O_4_, Fe_3_O_4_@Au-seed/DTNB, Fe_3_O_4_@Au/DTNB, and Fe_3_O_4_@Au/DTNB/Au/4-MPBA MNPs were successfully synthesized (Figure 1i). X-ray mapping was conducted to confirm the basic element composition of Fe_3_O_4_@Au/DTNB/Au/4-MPBA, Fe and O elements were mainly distributed in the middle of MNPs, while Au element existed on the shell (Figure 1j).

The SERS activities of Fe_3_O_4_, Fe_3_O_4_@Au-seed/DTNB, and Fe_3_O_4_@Au/DTNB were tested, the feature peaks of DTNB at 736, 826, 1061, 1149, 1331, and 1557 cm^−1^ were detected, among them, 1331 cm^−1^ was the strongest peak (Figure 1k) and the corresponding enhancement factor (EF) was 1.3 × 10^6^ (S4 and Figure S2), therefore, 1331 cm^−1^ was recognized as the characteristic peak for further analysis. The ability of Fe_3_O_4_@Au (without modification of DTNB) to enhance Raman signal and act as SERS were also validated using conventional dyes CV (S3 and Figure S1). As shown in Figure 1l, the zeta potential of Fe_3_O_4_ was −13.0 mV, the positively charged PEI and negatively charged 3-nm AuNPs were decorated onto the surface of Fe_3_O_4_ under electrostatic interaction, zeta potentials of Fe_3_O_4_−PEI and Fe_3_O_4_@Au-seed were +27.2 mV and +6.7 mV, respectively, while the potential of Fe_3_O_4_@Au/DTNB was decreased to −21.2 mV owing to the reduction of HAuCl_4_. Similarly, the potential of Fe_3_O_4_@Au/DTNB/PEI was +39.4 mV and Fe_3_O_4_@Au/DTNB/Au was +6.7 mV after the second layer of PEI and 3-nm AuNPs were coated on Fe_3_O_4_@Au/DTNB, which confirmed that Fe_3_O_4_@Au/DTNB/Au was successfully fabricated [33,34].

### 3.3. Optimization of the Fe_3_O_4_@Au/DTNB/Au/4-MPBA-Based LFA Strip

**Figure 2:**
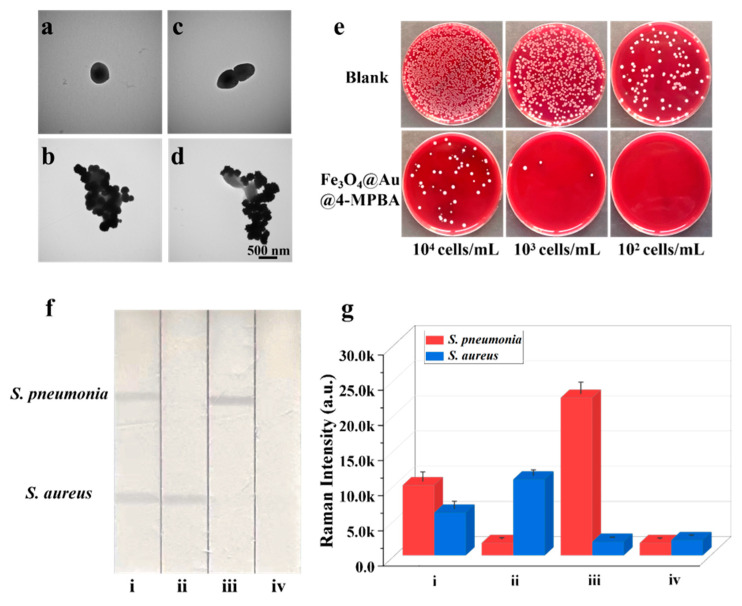
TEM images of (**a**) *S. aureus*, (**b**) *S. aureus* captured by Fe_3_O_4_@Au/DTNB/Au/4-MPBA, (**c**) *S. pneumonia*, and (**d**) *S. pneumonia* captured by Fe_3_O_4_@Au/DTNB/Au/4-MPBA. (**e**) Photographs of bacteria capture, the colonies on blood agar plates represent the amounts of remaining *S. aureus* and *S. pneumonia* in the supernatant after being captured by Fe_3_O_4_@Au/DTNB/Au/4-MPBA MNPs, the concentrations of bacteria ranged from 10^4^ cells mL^−1^ to 10^2^ cells mL^−1^. (**f**) Images and (**g**) corresponding SERS intensities of Fe_3_O_4_@Au/DTNB/Au/4-MPBA-LFA with different concentrations of *S. aureus* and *S. pneumonia*: (i) 10^5^, 10^5^ cells mL^−1^; (ii) 10^5^, 0 cells mL^−1^; (iii) 0, 10^5^ cells mL^−1^; and (iv) 0, 0 cells mL^−1^.

The Fe_3_O_4_@Au/DTNB/Au/4-MPBA tags can capture bacteria because 4-MPBA binds lipopolysaccharide that locates on the bacterial cell wall [35]. The TEM images clearly revealed that Fe_3_O_4_@Au/DTNB/Au/4-MPBA could effectively capture *S. aureus* and *S. pneumoniae* (Figure 2a–d). The bacteria capture efficiency was further determined by incubating Fe_3_O_4_@Au/DTNB/Au/4-MPBA in bacterial solutions of different concentrations. After magnetic enrichment of Fe_3_O_4_@Au/DTNB/Au/4-MPBA-bacteria complexes, the remaining bacteria in the supernatant were detected with traditional plate counting method and the capture efficiency of Fe_3_O_4_@Au/DTNB/Au/4-MPBA was about 95.4% for target bacteria from 10^4^ cells mL^−1^ to 10^2^ cells mL^−1^ (Figure 2e), demonstrating that Fe_3_O_4_@Au/DTNB/Au/4-MPBA SERS tags could capture almost all the bacteria in the sample solution.

Based on SERS strip, the specificity of antibodies was demonstrated by the solutions containing 10^5^ CFU mL^−1^ of *S. aureus*, *S. pneumoniae*, and their mixture. As displayed in Figure 2f, two test lines of *S. aureus* and *S. pneumoniae* on NC membrane could be observed, indicating that there were two target bacteria in the detection sample, whereas only one test line was observed for *S. aureus* or *S. pneumoniae* that existed alone. Additionally, the SERS intensity values of *S. aureus* or *S. pneumoniae* alone were higher than that of *S. aureus* and *S. pneumoniae* simultaneous existence owing to broad-spectrum capture performance of 4-MPBA (Figure 2g), indicating an excellent specificity capability of *S. aureus* and *S. pneumoniae* antibodies. Here, bacteria captured by Fe_3_O_4_@Au/DTNB/Au/4-MPBA had little effect on Fe_3_O_4_@Au/DTNB/Au/4-MPBA-LFA, the generation of signals were mainly attributed to Fe_3_O_4_@Au/DTNB/Au/4-MPBA (S5 and Figure S3).

Considering the detection performance of the system, several critical parameters were optimized and provided in the supporting information. The NC membranes of Prima40 (18 μm pore size), CN140 (8 μm pore size), and CN95 (15 μm pore size) were evaluated using 10^6^ CFU mL^−1^ of *S. aureus* and *S. pneumoniae.* The signal-to-noise ratio (SNR) was calculated by the ratio of the SERS signal intensities of positive and negative samples, CN95 membrane displayed the highest SNR for target bacteria (Figure S4). Owing to the immunobinding between Fe_3_O_4_@Au/DTNB/Au/4-MPBA-bacteria complexes and antibodies, the running buffer was further optimized, the results confirmed that PBS solution containing 2% (*w*/*v*) Tween and 15% (*w*/*v*) FBS could suppress the non-specific combination of Fe_3_O_4_@Au/DTNB/Au/4-MPBA on the test lines and obtained maximized SNR for target bacteria (Figure S5). A serial usage of Fe_3_O_4_@Au/DTNB/Au/4-MPBA tags 1 μL, 2 μL, 4 μL, 6 μL, and 8 μL were respectively incubated with *S. aureus* and *S. pneumoniae* at room temperature, the results showed that 4 μL of tags provided the highest SNR for target bacteria detection (Figure S6). Antibody concentrations of target bacteria were also optimized, 0.4 mg mL^−1^ of *S. pneumoniae* antibody and 0.6 mg mL^−1^ of *S. aureus* antibody could provide the highest SNR of SERS intensities (Figure S7). Finally, the reaction time was optimized, SNR increased from 0 to 20 min and then slightly lower after 20 min (Figure S8). Therefore, the reaction time of 20 min was enough for the whole process.

### 3.4. Analytical Performance of Fe_3_O_4_@Au/DTNB/Au/4-MPBA-Based LFA

**Figure 3:**
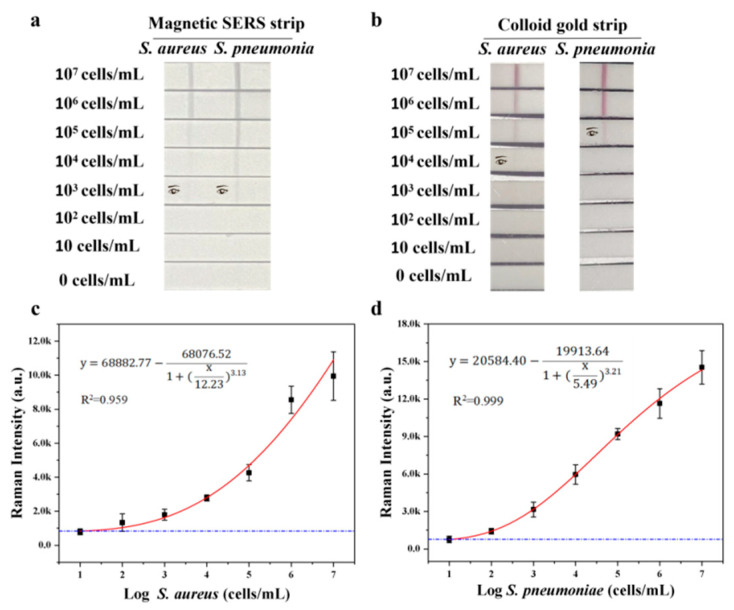
(**a**) Photographs of Fe_3_O_4_@Au/DTNB/Au/4-MPBA-based LFA strips for the detection at different concentrations of target bacteria. The visualization concentrations of target bacteria are both 10^3^ cells mL^−1^. (**b**) Photographs of colloidal gold-based LFA strips for target bacteria detection. The visualization concentrations of *S. aureus* and *S. pneumoniae* are 10^4^ and 10^5^ cells mL^−1^ for AuNP−LFA strip detection, respectively. (**c**,**d**) Corresponding calibration curves of target bacteria for Fe_3_O_4_@Au/DTNB/Au/4-MPBA-based LFA strips.

To access the sensitivity and selectivity of our system, different concentrations of *S. aureus* and *S. pneumoniae* from 0 to 10^7^ CFU mL^−1^ were mixed with Fe_3_O_4_@Au/DTNB/Au/4-MPBA tags and then detected by Fe_3_O_4_@Au/DTNB/Au/4-MPBA-LFA strips. As shown in Figure 3a, with the decreasing concentration of target bacteria, the color of the test lines gradually shallowed. It could be observed with the naked eyes that the visualization concentrations of target bacteria were both 10^3^ cells mL^−1^, respectively. Moreover, the SERS signal intensities of test lines at 1331 cm^−1^ increased with increasing bacterial concentration, the calibration curves of two test lines displayed dynamic relationships between SERS intensities and the concentrations of target bacteria and the correlation coefficients (R^2^) were 0.959 and 0.999 for *S. aureus* and *S. pneumoniae*, respectively (Figure 3c,d). Based on the calculation of blank SERS signal, LODs of Fe_3_O_4_@Au/DTNB/Au/4-MPBA were determined to be 12 and 11 cells mL^−1^ for *S. aureus* and *S. pneumoniae*, respectively, according to the International Union of Pure and Applied Chemistry (IUPAC) protocol (LOD = mean SERS signal intensities of blank groups + 3 × standard deviation of the control) [36]. We compared our method with standard colloidal gold (AuNP)-LFA method, the preparation of colloidal AuNPs was displayed in supporting information S2, the visualization concentrations of *S. aureus* and *S. pneumoniae* were 10^4^ and 10^5^ cells mL^−1^ for AuNP-LFA strip detection, respectively (Figure 3b). Therefore, our detection system Fe_3_O_4_@Au/DTNB/Au/4-MPBA-LFA presented excellent sensitivity.

**Figure 4:**
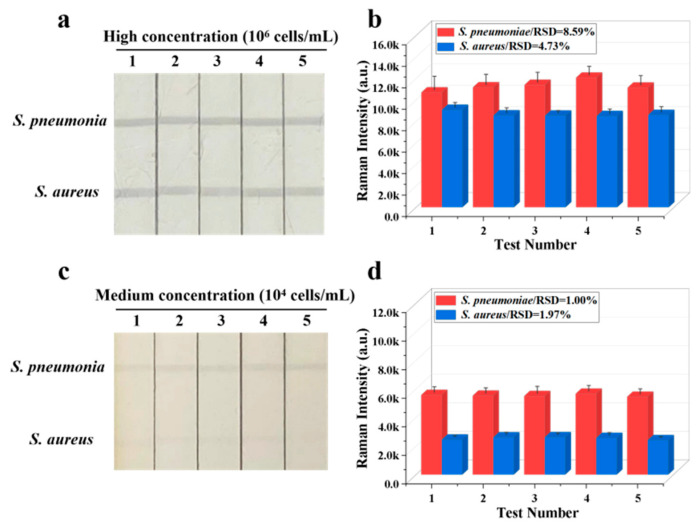
Reproducibility of Fe_3_O_4_@Au/DTNB/Au/4-MPBA-LFA strips for target bacteria at concentrations of (**a**) 10^6^ and (**c**) 10^4^ cells mL^−1^, respectively, and corresponding SERS intensities (**b**,**d**) on the test lines.

**Figure 5:**
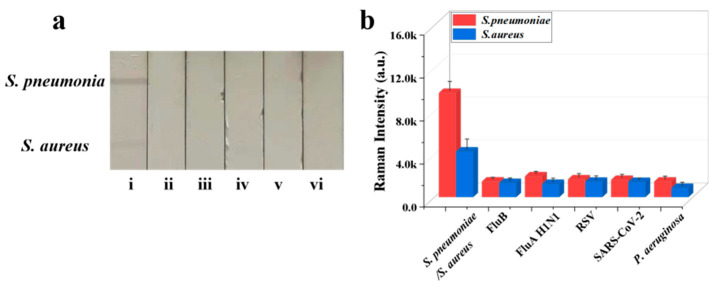
(**a**) Specificity of Fe_3_O_4_@Au/DTNB/Au/4-MPBA-LFA strips for target bacteria at concentration of 10^5^ cells mL^−1^: (i) *S. pneumoniae/S. aureus*; (ii) FluB; (iii) FluA H1N1; (iv) RSV; (v) SARS-CoV-2; and (vi) *P. aeruginosa.* (**b**) Corresponding SERS intensities for *S. aureus*, *S. pneumoniae*, and other negative samples.

The reproducibility of Fe_3_O_4_@Au/DTNB/Au/4-MPBA-based LFA system is a crucial factor for its practical application. The bacteria *S. pneumoniae* and *S. aureus* were diluted into a high-dose group of 10^6^ CFU mL^−1^, the results indicated good reproducibility with relative standard deviation (RSD) of 8.59% and 4.73% for *S. pneumoniae* and *S. aureus,* respectively (Figure 4a,b). The diluted medium-dose group of 10^4^ CFU mL^−1^ was also conducted with RSD of 1.00% and 1.97% for *S. pneumoniae* and *S. aureus,* respectively (Figure 4c,d). The specificity of Fe_3_O_4_@Au/DTNB/Au/4-MPBA-based LFA was further evaluated, several respiratory viruses including FluB (10^5^ copies mL^−1^), FluA H1N1 (10^5^ copies mL^−1^), RSV (10^5^ pfu mL^−1^), SARS-CoV-2 (100 ng mL^−1^), and respiratory bacteria *P. aeruginosa* (10^6^ CFU mL^−1^) were used as negative samples [37,38], as displayed in Figure 5a,b, the positive groups containing target bacteria generated higher SERS intensities on their corresponding test lines, whereas all the negative samples displayed low SERS signal intensities, confirming Fe_3_O_4_@Au/DTNB/Au/4-MPBA-based LFA possessed good specificity. Finally, the recovery rates of Fe_3_O_4_@Au/DTNB/Au/4-MPBA-LFA strips were tested in the sputum specimens, as shown in Table 1, the average recoveries for *S. aureus* and *S. pneumoniae* ranged from 92.7% to 111.5%, and the RSD values of the SERS intensities on two test lines were less than 10%, therefore, Fe_3_O_4_@Au/DTNB/Au/4-MPBA-LFA strip had potential to detect *S. aureus* and *S. pneumoniae* in the real sputum specimens.

## 4. Conclusions

In this work, we fabricated a novel strip based on Fe_3_O_4_@Au/DTNB/Au/4-MPBA SERS nanotags for simultaneous detection of two respiratory bacteria. The SERS tags possessed magnetic enrichment and broad-spectrum bacteria capture capabilities. Our results showed that Fe_3_O_4_@Au/DTNB/Au/4-MPBA-LFA biosensor was ultrasensitive and the LODs of *S. aureus* and *S. pneumoniae* were as low as 12 and 11 CFU mL^−1^, respectively. Moreover, the biosensor displayed good reproducibility, excellent specificity, and high recovery rates in the sputum specimens. If the antibodies on NC membrane are altered, the strip can be used to detect other specific bacteria. Thus, the proposed multifunctional tool provided a universal platform for the detection of respiratory pathogens.

## Figures and Tables

**Table 1:** Results of the recovery test in the sputum specimens (*n* = 3).

Strain	Spiked (cells/mL)	Detected (cells/mL)	Recovery (%)	RSD (%)
*S. pneumoniae*	1 × 10^6^	9.27 × 10^5^	92.7	3.04
	1 × 10^3^	1.115 × 10^3^	111.5	8.59
*S. aureus*	1 × 10^6^	1.065 × 10^6^	106.5	6.22
	1 × 10^3^	1.04 × 10^3^	104.2	8.26

## Data Availability

Not applicable.

## Supplementary Materials

The following supporting information [39,40] can be downloaded at: https://www.mdpi.com/article/10.3390/bios13020210/s1, S1. Preparation of Fe_3_O_4_@Au/DTNB; S2. Preparation of Colloidal AuNPs; S3. Evaluation of the Surface Enhancement Ability of Fe_3_O_4_@Au; S4. Calculation of Enhancement Factor (EF) for the SERS Detection; S5. Raman Spectra of *S. aureus* and *S. pneumoniae* with and without the Use of LFA.
