# Correction: Li et al. Magnetic SERS Strip Based on 4-mercaptophenylboronic Acid-Modified Fe_3_O_4_@Au for Active Capture and Simultaneous Detection of Respiratory Bacteria. *Biosensors* 2023, *13*, 210

**DOI:** 10.3390/bios16020091

**Published:** 2026-02-02

**Authors:** Jingfei Li, Jin Chen, Yuwei Dai, Zhenzhen Liu, Junnan Zhao, Shuchen Liu, Rui Xiao

**Affiliations:** 1 Beijing Institute of Microbiology and Epidemiology, Beijing 100071, China; ljf13100960516@163.com (J.L.); chenjin18896650390@163.com (J.C.); dywbetter@163.com (Y.D.); liuz101102@163.com (Z.L.); zhaojunnan1997@163.com (J.Z.); 2 Department of Clinical Laboratory, Beijing Ditan Hospital, Capital Medical University, Beijing 100015, China; 3 Beijing Institute of Radiation Medicine, Beijing 100850, China

## Figure/Table Correction

Due to an error in the original publication [1], a correction has been made in Scheme 1, “Absorbing line” has been changed to “Absorbing pad”, and the position of the compounds on test line 1 (below, *S. aureus*) and test line 2 (above, *S. pneumoniae*) has been changed. There was an error in Figure 3 as published due to an error when the paper was uploaded. In view of the subtle changes in the sensitivity results, in order to be consistent with the sensitivity, we have made corresponding changes to the reproducibility, specificity, and recovery rate experiments. The corrected figures and table appear below.

**Scheme 1:**
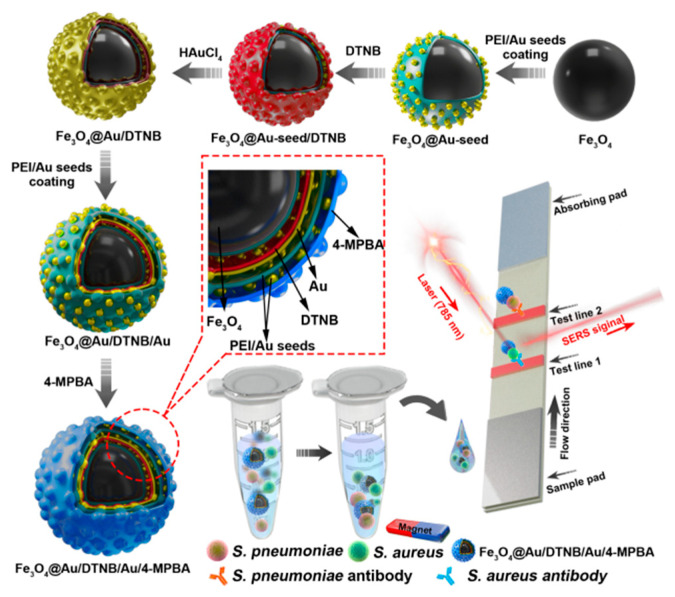
Fabrication of Fe_3_O_4_@Au/DTNB/Au/4-MPBA and schematic diagram of Fe_3_O_4_@Au/DTNB/Au/4-MPBA SERS strip for detecting two respiratory bacteria.

**Figure 3:**
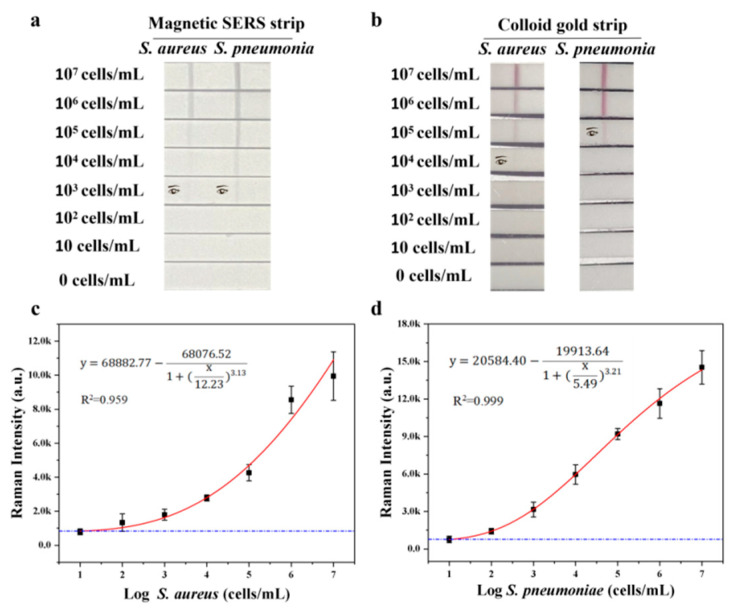
(**a**) Photographs of Fe_3_O_4_@Au/DTNB/Au/4-MPBA-based LFA strips for the detection at different concentrations of target bacteria. The visualization concentrations of target bacteria are both 10^3^ cells mL^−1^. (**b**) Photographs of colloidal gold-based LFA strips for target bacteria detection. The visualization concentrations of *S. aureus* and *S. pneumoniae* are 10^4^ and 10^5^ cells mL^−1^ for AuNP−LFA strip detection, respectively. (**c**,**d**) Corresponding calibration curves of target bacteria for Fe_3_O_4_@Au/DTNB/Au/4-MPBA-based LFA strips.

**Figure 4:**
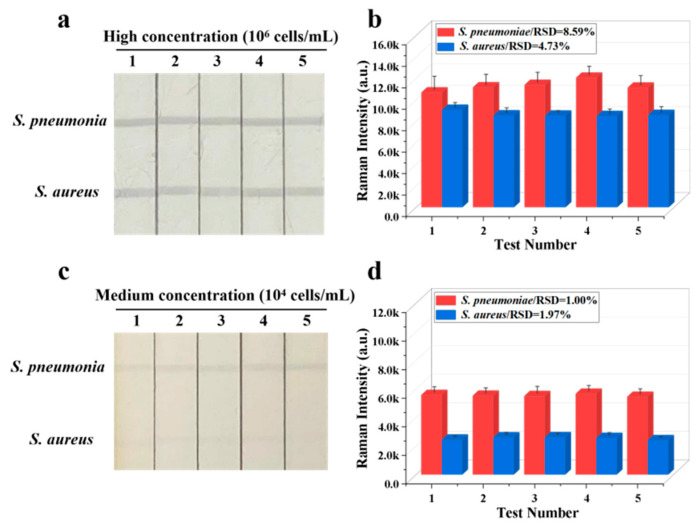
Reproducibility of Fe_3_O_4_@Au/DTNB/Au/4-MPBA-LFA strips for target bacteria at concentrations of (**a**) 10^6^ and (**c**) 10^4^ cells mL^−1^, respectively, and corresponding SERS intensities (**b**,**d**) on the test lines.

**Figure 5:**
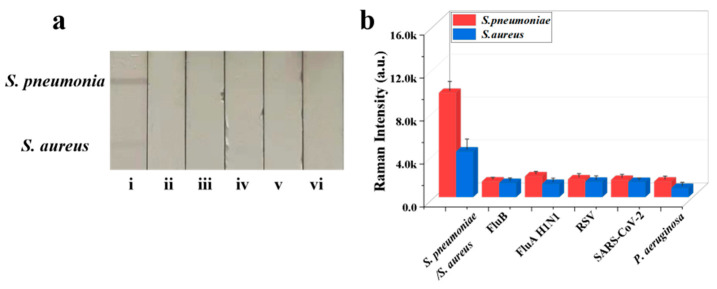
(**a**) Specificity of Fe_3_O_4_@Au/DTNB/Au/4-MPBA-LFA strips for target bacteria at concentration of 10^5^ cells mL^−1^: (i) *S. pneumoniae*/*S. aureus*; (ii) FluB; (iii) FluA H1N1; (iv) RSV; (v) SARS-CoV-2; and (vi) *P. aeruginosa*. (**b**) Corresponding SERS intensities for *S. aureus*, *S. pneumoniae*, and other negative samples.

**Table 1:** Results of the recovery test in the sputum specimens (*n* = 3).

Strain	Spiked (cells/mL)	Detected (cells/mL)	Recovery (%)	RSD (%)
*S. pneumoniae*	1 × 10^6^	9.27 × 10^5^	92.7	3.04
	1 × 10^3^	1.115 × 10^3^	111.5	8.59
*S. aureus*	1 × 10^6^	1.065 × 10^6^	106.5	6.22
	1 × 10^3^	1.04 × 10^3^	104.2	8.26

## Text Correction

There were some errors in the original publication. The limits of detection, correlation coefficients, and relative standard deviation need to be corrected. The corrections are as follows:

Abstract

“the limits of detection for *S. aureus* and *S. pneumoniae* were as low as 8 and 13 CFU mL^−1^, respectively” is replaced by “the limits of detection for *S. aureus* and *S. pneumoniae* were as low as 12 and 11 CFU mL^−1^, respectively”.

1. Introduction

Paragraph 4: “as low as 8 and 13 CFU mL^−1^” is replaced by “as low as 12 and 11 CFU mL^−1^”.

3. Results and Discussion

3.4. Analytical Performance of Fe_3_O_4_@Au/DTNB/Au/4-MPBA-Based LFA

Paragraph 1: “were 10 and 10^3^ cells mL^−1^” is replaced by “were both 10^3^ cells mL^−1^”; “the correlation coefficients (R^2^) were 0.994 and 0.995” is replaced by “the correlation coefficients (R^2^) were 0.959 and 0.999”; and “8 and 13 cells mL^−1^ for *S. aureus* and *S. pneumoniae*” is replaced by “12 and 11 cells mL^−1^ for *S. aureus* and *S. pneumoniae*”.

Paragraph 2: “relative standard deviation (RSD) of 6.32% and 5.12%” is replaced by ”relative standard deviation (RSD) of 8.59% and 4.73%”; “RSD of 9.43% and 8.63%” is replaced by “RSD of 1.00% and 1.97%”; and “ranged from 92.3% to 105.2%” is replaced by “ranged from 92.7% to 111.5%”.

4. Conclusions

“as low as 8 and 13 CFU mL^−1^” is replaced by “as low as 12 and 11 CFU mL^−1^”.

Furthermore, in the original publication, the word “cells” is missing in the caption of Figure 2. A correction “(iv) 0, 0 cells mL^−1^” has been made to the caption of Figure 2.
