# Supplementary material for: Magnetic SERS Strip Based on 4-mercaptophenylboronic Acid-Modified Fe3O4@Au for Active Capture and Simultaneous Detection of Respiratory Bacteria
Source: Biosensors (Basel). 2023 Jan 31;13(2):210. doi: 10.3390/bios13020210 (PMC9953780; doi:10.3390/bios13020210)
Supplement: Supplementary file 1 [file biosensors-13-00210-s001.zip › biosensors-2126826-supplementary.pdf]

biosensors

Supporting Information

Magnetic SERS Strip Based on 4-mercaptophenylboronic Acid-Modified Fe_3O_4@Au for Active Capture and Simultaneous Detection of Respiratory Bacteria

Jingfei Li [1,†], Jin Chen [1,3,†], Yuwei Dai [1], Zhenzhen Liu [1], Junnan Zhao [1] and Shuchen Liu [2,*] and Rui Xiao [1,*]

1 Beijing Institute of Microbiology and Epidemiology, Beijing, China
2 Beijing Institute of Radiation Medicine, Beijing 100850, China
3 Department of Clinical Laboratory, Beijing Ditan Hospital, Capital Medical University, Beijing 100015
* Correspondence: liusc118@163.com (S.L.); ruixiao203@163.com (R.X.)
† These authors contributed equally to this work.

S1. Preparation of Fe_3O_4@Au/DTNB

Fe_3O_4@Au/DTNB was synthesized using a PEI−mediated seed growth method. First, the Fe_3O_4 nanoparticles (200 nm in diameter) were synthesized according to a previously reported method [1]. Second, 0.1 g of prepared Fe_3O_4 nanoparticles were dispersed in the PEI aqueous solution (2 mg/mL) under sonication for 30 min, during which the PEI gradually self-assembled on the surface of the Fe_3O_4. After washing twice, the Fe_3O_4−PEI was mixed with colloidal 3-nm AuNPs and sonicated for 30 min to form Fe_3O_4@Au−seed. Then, 15 μL of DTNB (10 mM) was added to 5 mL of Fe_3O_4@Au−seed ethanolic solution and the mixture was sonicated for about 1.5 h to fabricate Fe_3O_4@Au−seed/DTNB. Finally, 1 mL of Fe_3O_4@Au seed/DTNB was dispersed in 40 mL of aqueous solution containing 3% (w/v) PVP and 0.25 mM $HAuCl_4$ followed by sonication for 30 min to obtain Fe_3O_4@Au/DTNB. The Fe_3O_4@Au/DTNB was enriched and stored at 5 mL ethanol solution for further use.

S2. Preparation of Colloidal AuNPs

First, colloidal AuNPs (40 nm in diameter) were prepared according to the classical sodium citrate reduction method [2]. Second, *S. pneumoniae* or *S. aureus* (5 μg) antibody was incubated with 1 mL of AuNPs for 1 h. Then, BSA with a concentration of 10 mg/mL (50 μL) was added to block the unreacted sites of AuNPs. The as-prepared immuno−AuNPs were separated using centrifugation (4000 rpm, 7 min) and resuspended with 100 μL of storage solution (2 mM PB solution). Finally, the antibody−conjugated AuNPs were directly mixed with the bacteria solution and the prepared strip was inserted into the solution to have a chromatographic reaction.

S3. Evaluation of the Surface Enhancement Ability of Fe_3O_4@Au

An appropriate concentration of the Fe_3O_4@Au solution, CV solution, and their mixed solution Fe_3O_4@Au/CV were dropped on the Si chip; then, a portable Raman spectrometer was used to detect the Raman signals. The obvious peaks for Fe_3O_4@Au were not observed, while CV displayed very weak characteristic peaks that were strengthened after the CV mixed with the Fe_3O_4@Au.

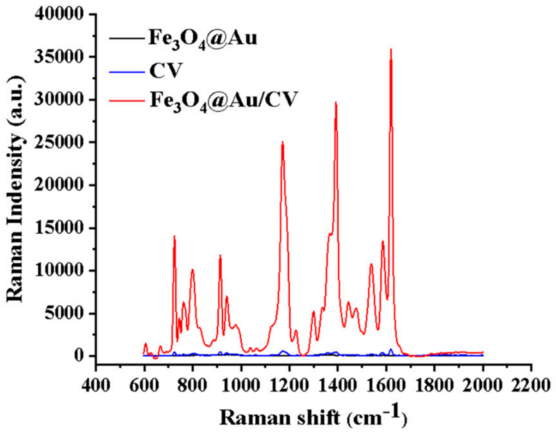

Figure S1. SERS signal intensities of Fe_3O_4@Au, CV, and Fe_3O_4@Au/CV.

S4. Calculation of Enhancement Factor (EF) for the SERS Detection

$EF = \frac{I_{SERS}}{N_{SERS}}\frac{N_{bulk}}{I_{bulk}}$, where I_{SERS} and I_{bulk} are the SERS intensities of the related peaks of measured molecules with and without substrate enhancement, respectively. N_{SERS} and N_{bulk} are the average number of measured molecules. However, the actual EF cannot be obtained easily because it is difficult to measure some important parameters such as the number of measured molecules and the projection depth of the laser. Notably, when other test conditions are consistent, the concentration of measured molecules and their number are in positive proportion under the same loading volume. Therefore, in our experiment, the EF value can be roughly estimated according to the equation: $EF = \frac{I_{SERS}}{I_{RS}}\frac{C_{RS}}{C_{SERS}}$, where I_{SERS} and I_{RS} are the SERS intensities of characteristic peak of DTNB in Fe_3O_4@Au and pure DTNB ethanol solution, respectively. C_{SERS} and C_{RS} are the concentrations of DTNB modified on Fe_3O_4@Au and ethanol, respectively. The Raman intensity at 1331 cm^{-1} of DTNB modified on Fe_3O_4@Au MNPs (3×10^{-6} M) and DTNB (0.1 M) were 32176.02 a.u. and 809.47 a.u., respectively. Therefore, $EF_{1331\ cm^{-1}} = \frac{I_{SERS}}{I_{RS}}\frac{C_{RS}}{C_{SERS}} = \frac{32176.02}{809.47} \times \frac{0.1}{3\times10^{-6}}$ =1.3×10^6.

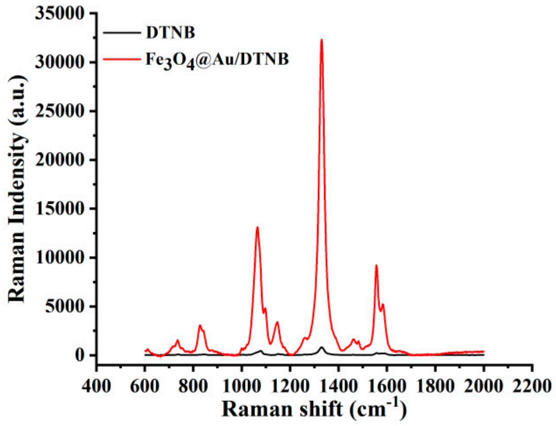

Figure S2. Raman spectra of DTNB and DTNB@Au/DTNB.

S5. Raman Spectra of *S. aureus* and *S. pneumoniae* with and without the Use of LFA

An appropriate concentration of *S. aureus* solution and *S. pneumoniae* solution were dropped on the Si chip, then SERS signals were detected using a portable Raman spectrometer under 785 nm laser excitation. Meanwhile, for the signal measurement using

Fe_3O_4@Au/DTNB/Au/4–MPBA–LFA, *S. aureus* and *S. pneumonia* with a concentration of 10^6 CFU mL^{-1} were suspended in 80 μL of PBS running buffer solution, the prepared strip was inserted into a PBS running buffer solution for 20 min, and then the strip was dried and the SERS intensities of the test lines on NC membrane were measured using the Raman spectrometer. The results showed that no characteristic peak was observed at 1331 cm^{-1}, while there was a strong peak characteristic peak at 1331 cm^{-1} using Fe_3O_4@ Au/DTNB/Au/4–MPBA–LFA and the peak values were much higher than that of bacteria , indicating that bacteria had little ef-fect on the peak value.

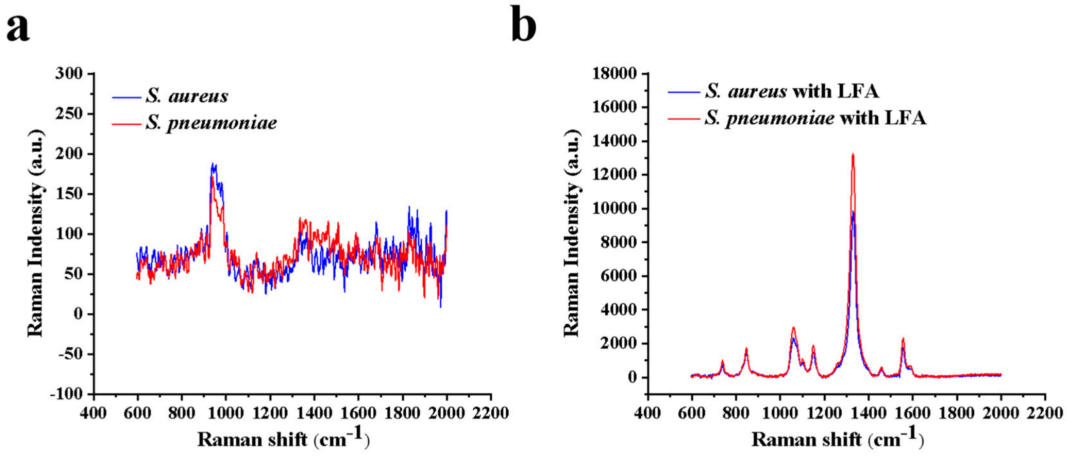

Figure S3. (**a**) SERS signal intensities of *S. aureus* and *S. pneumoniae*, (**b**) SERS signal intensities of *S. aureus* with LFA and *S. pneumoniae* with LFA.

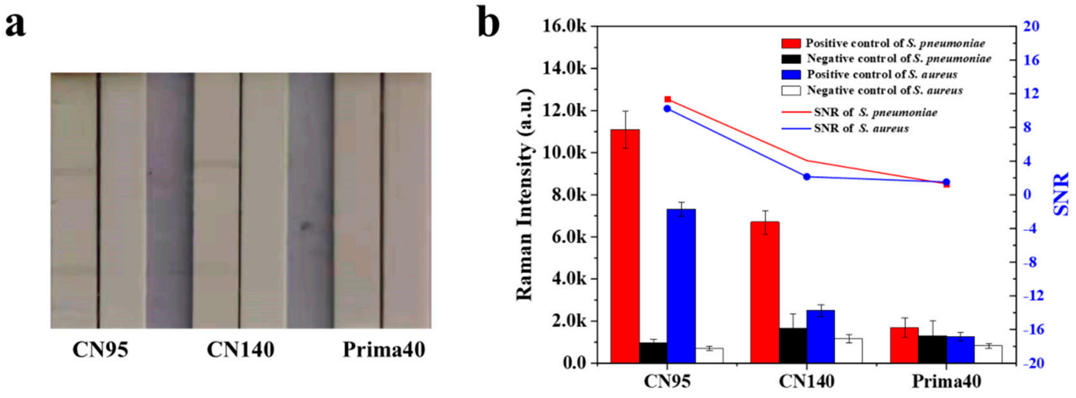

Figure S4. Optimization of NC membrane for Fe_3O_4@Au/DTNB/Au/4–MPBA–LFA strip. Photographs (**a**) and corresponding Raman intensities (**b**) of Fe_3O_4@Au/DTNB/Au/4–MPBA–LFA strips at different NC membranes. The error bars indicate standard deviations calculated from five measurements.

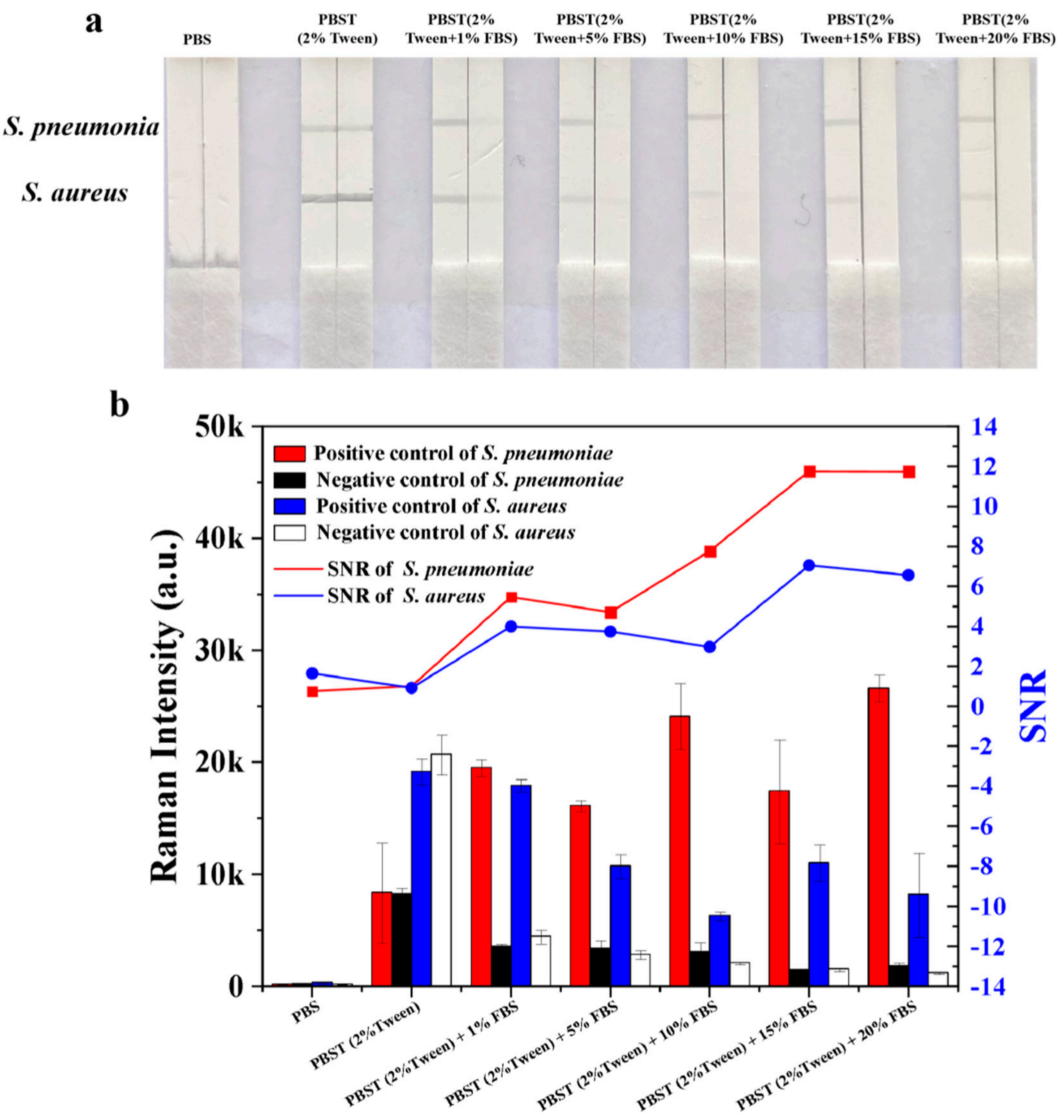

Figure S5. Optimization of running buffer for Fe_3O_4@Au/DTNB/Au/4–MPBA–LFA strip. Photographs (**a**) and corresponding Raman intensities (**b**) of Fe_3O_4@Au/DTNB/Au/4–MPBA–LFA strips at different running buffer solutions. The error bars indicate standard deviations calculated from five measurements.

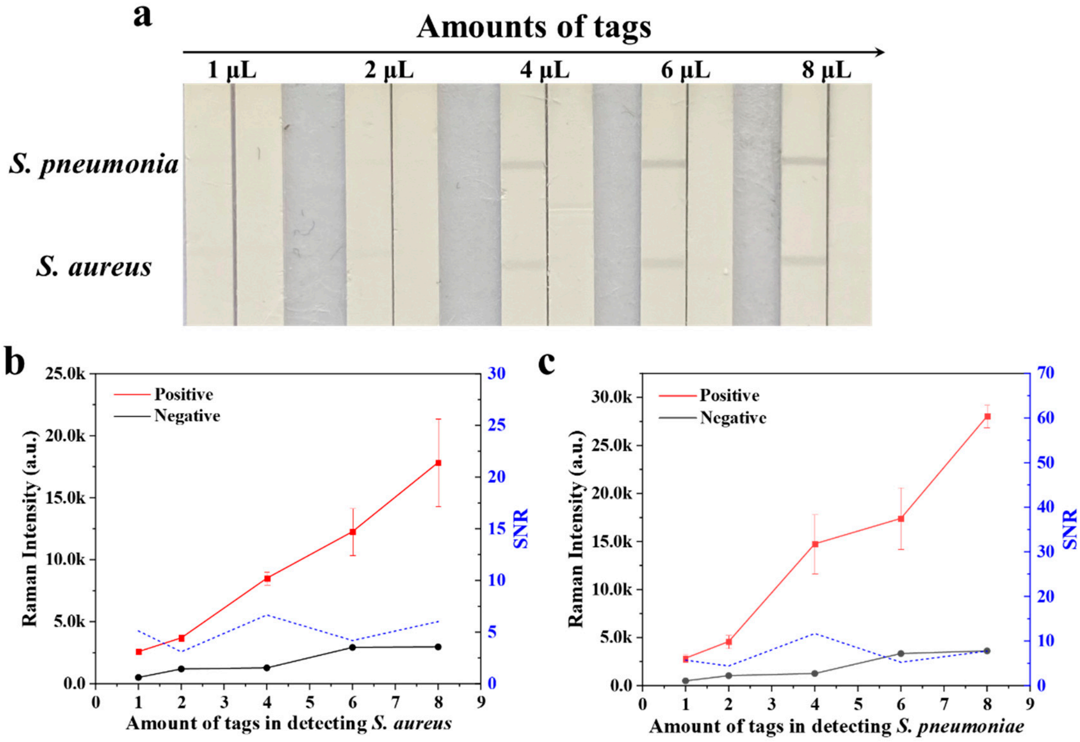

Figure S6. Optimization of tag amounts for Fe_3O_4@Au/DTNB/Au/4−MPBA−LFA strip. Photographs of Fe_3O_4@Au/DTNB/Au/4−MPBA−LFA strips with different tag amounts (**a**). Corresponding Raman intensities on test lines for *S. aureus* (**b**) and *S. pneumoniae* (**c**), respectively. The error bars indicate standard deviations calculated from five measurements.

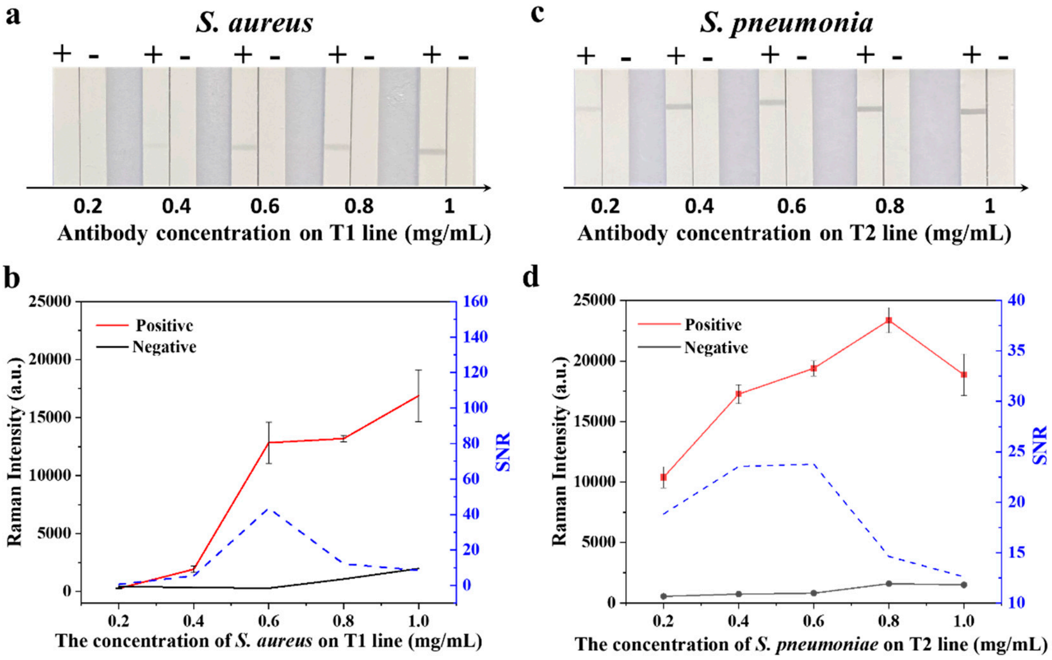

Figure S7. Optimization of antibody concentration for Fe_3O_4@Au/DTNB/Au/4−MPBA−LFA strip. Photographs (**a**,**c**) and corresponding Raman intensities at different *S. aureus* (**b**) and *S. pneumoniae* (**d**) antibody concentrations on test lines. The error bars indicate standard deviations calculated from five measurements.

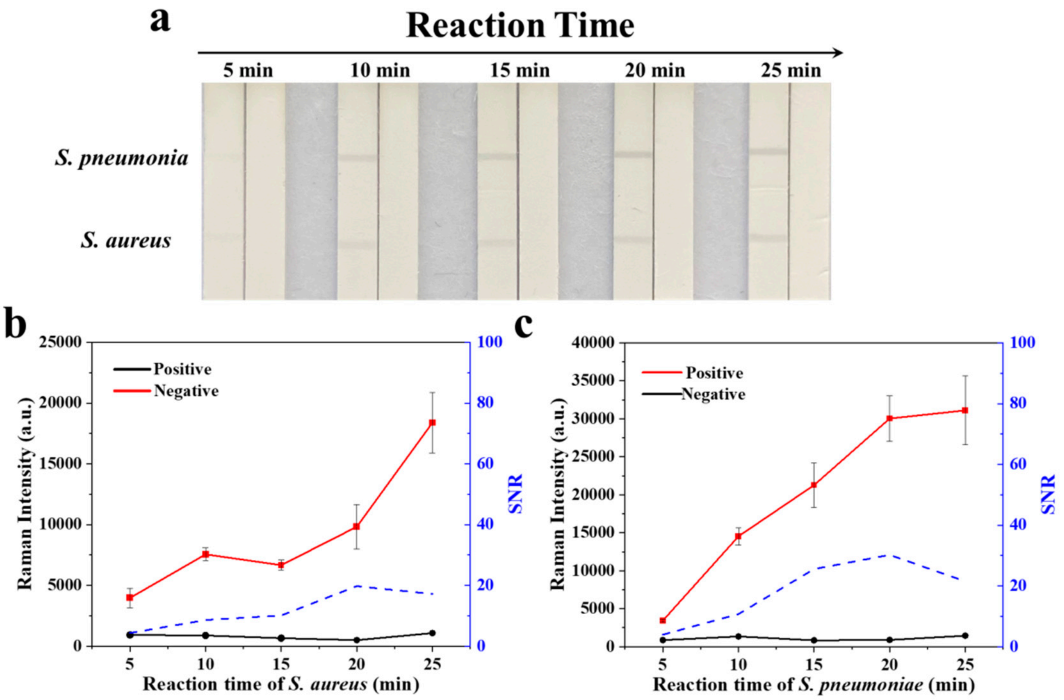

Figure S8. Optimization of reaction time for Fe_3O_4@Au/DTNB/Au/4−MPBA−LFA strip. Photographs (**a**) and the corresponding Raman intensities of test lines at different reaction time for *S. aureus* (**b**) and *S. pneumoniae* (**c**), respectively. The error bars indicate standard deviations calculated from five measurements.

References

1. Liu, X.; Yang, X.; Li, K.; Liu, H.; Xiao, R.; Wang, W.; Wang, C.; Wang, S. Fe_3O_4@Au SERS tags-based lateral flow assay for simultaneous detection of serum amyloid A and C-reactive protein in unprocessed blood sample. *Sens Actuators B Chem*, **2020**, *320*, 128350.
2. Li, J.F.; Tian, X.D.; Li, S.B; Anema, J.R.; Yang, Z.L.; Ding, Y.; Wu, Y.F.; Zeng, Y.M.; Chen, Q.Z.; Ren, B.; et al. Surface analysis using shell-isolated nanoparticle-enhanced Raman spectroscopy. *Nat Protoc*, **2013**, *8*, 52–65.
